# Ebola Risk Perception in Germany, 2014

**DOI:** 10.3201/eid2106.150013

**Published:** 2015-06

**Authors:** Nicole Rübsamen, Stefanie Castell, Johannes Horn, André Karch, Jördis J. Ott, Heike Raupach-Rosin, Beate Zoch, Gérard Krause, Rafael T. Mikolajczyk

**Affiliations:** Helmholtz Centre for Infection Research, Braunschweig, Germany (N. Rübsamen, S. Castell, J. Horn, A. Karch, J.J. Ott, H. Raupach-Rosin, B. Zoch, G. Krause, R.T. Mikolajczyk);; PhD Programme “Epidemiology,” Braunschweig-Hannover, Germany (N. Rübsamen, J. Horn, A. Karch, B. Zoch);; German Centre for Infection Research, Hannover-Braunschweig, Germany (A. Karch, J.J. Ott, G. Krause, R.T. Mikolajczyk);; Hannover Medical School, Hannover, Germany (G. Krause, R.T. Mikolajczyk)

**Keywords:** behavior, hemorrhagic fever, Ebola, risk, perception, transmission, viruses, Germany, Ebola virus, Ebola virus disease, EVD

## Abstract

Ebola virus disease (EVD) outbreaks have occurred during the past 5 decades, but none has affected European countries like the 2014 epidemic in West Africa. We used an online questionnaire to investigate risk perceptions in Germany during this epidemic peak. Our questionnaire covered risk perceptions, knowledge about transmission routes, media use, reactions to the outbreak, attitudes toward measures to prevent the spread of EVD and vaccination against EVD, and willingness to volunteer for aid missions. Of 974 participants, 29% indicated that they worried about EVD, 4% correctly stated virus transmission routes, and 75% incorrectly rated airborne transmission and transmission by asymptomatic patients as possible. Many indicated that if a patient were flown to Germany for treatment in a nearby hospital, they would adapt preventive behavior. Although most participants were not worried about EVD at the current stage of the epidemic, misperceptions regarding transmission were common and could trigger inappropriate behavior changes.

## Abstract 2

Knowledge about actual risks was poor, creating the potential for inappropriate behavior changes.

Misperceptions of risk can lead to inappropriate reactions during epidemics (*1*,*2*), such as stigmatization of those who are perceived as possible sources of infection (*3*). With regard to Ebola virus disease (EVD) in the West African countries most affected by the outbreak in 2014, indications are strong that societal misperceptions contributed to the outbreak spread (*4*). Public perceptions even in countries not directly affected by the EVD outbreak might influence outbreak response (e.g., by the priorities governments will set or by the willingness of persons to volunteer for aid missions in the affected countries) (*5*,*6*).

National authorities in countries outside of Africa responded differently to the potential risks of importing EVD into their countries. In November 2014, Australia and Canada imposed entry restrictions for persons from Guinea, Sierra Leone, and Liberia (*7*,*8*). At the same time, the United Kingdom introduced entry screening for international flight and train passengers (*9*,*10*). Because evidence for these public health actions is difficult to evaluate (*11*,*12*), public opinions might have played a role in political decision making. As of November 2014, Germany had not implemented any travel or entry restriction. As of October 14, 2014, a total of 3 patients who had acquired EVD in West Africa have been evacuated to hospitals in Germany for treatment. These evacuations to Germany were intensively covered by the media in Germany.

Several previous EVD outbreaks have occurred, but none was comparable in size and spread to the 2014 epidemic in West Africa and none directly or indirectly affected European countries, until now. To understand public reactions during an emerging epidemic in a country not directly affected by EVD, but one that is exposed to media coverage of the epidemic and involved in actions to contain the epidemic, we conducted an online survey about EVD for residents of Lower Saxony, Germany. Our goal was to improve our understanding of risk perceptions and potential changes in behavior during epidemics.

## Methods

### Participants

We implemented this survey by using a longitudinal online panel, which was created in March 2014 to address human hygiene and preventive behavior regarding infectious diseases (*13*,*14*). The panel consists of 1,376 persons 15–69 years of age, who complete short, online questionnaires once a month. Panel members come from 4 districts in Lower Saxony, Germany (Braunschweig, Salzgitter, Vechta, and Wolfenbüttel). The districts were chosen by convenience: Braunschweig is the location of our research institute (the Helmholtz Centre for Infection Research), Vechta is its rural counterpart, and Salzgitter and Wolfenbüttel are 2 neighboring districts of Braunschweig. In each district, potential participants were invited to the panel by means of proportional stratified random sampling from the population registry. Of 26,895 invited, 9% were successfully recruited. 

### Questionnaire

We used an open-source online survey application (Limesurvey; *15*) to develop a knowledge-attitude-practice survey for online use (*16*). In November 2014, the questionnaire was about EVD (Technical Appendix). The EVD questionnaire consisted of 27 questions with 2–11 items each, totaling 123 items. The questions covered 7 topics: worries about EVD and perceived personal probability of infection, knowledge about transmission routes of Ebola virus, media use to obtain information about EVD, personal reactions to the EVD outbreak, attitudes toward specific measures to prevent the spread of EVD to Europe, willingness to volunteer to fight EVD in West Africa, and attitudes toward vaccination against EVD.

Risk perceptions were operationalized by asking participants if they worry about EVD (“yes” or “no”) and how they perceive their personal probability of acquiring EVD in the following 9 scenarios: at work, in public transport, in public places, at an airport in Germany, as a patient in a hospital in Germany, at a doctor's office in Germany, during travel to affected countries, by food imported from West African countries, or by other products originating in West Africa. Responses were chosen from a Likert scale with 5 options: “yes,” “rather yes,” “rather no,” “no,” and “does not apply.” “Worry about EVD” describes thinking about threatening scenarios in the absence of actual danger (*17*), and “perceived personal probability of infection” describes participants’ estimation of the actual risk for infection.

Knowledge about the transmission of Ebola virus was assessed with regard to the following 11 potential transmission routes: by direct contact with bodily fluids of infected persons, dead or living; through material heavily contaminated with such fluids; by direct contact with infected but asymptomatic persons; through air; through material that has been heavily contaminated with bodily fluids of infected persons, dead or living; through drinking water; through food produced in Germany; by casual contact with someone already sick, such as sitting next to someone (and without any direct contact of bodily fluids); by wild animals in Africa; by insects in Africa; or by wild animals/insects in Germany. Response choices were “true,” “false,” and “don’t know.” We computed a cumulative knowledge score (1 point for each answer in agreement with current scientific knowledge, range 0–11). In addition, participants were invited to rate their personal knowledge of EVD as “very good,” “good,” “moderate,” or “not good.” They were also asked whether they increased their use of media to inform themselves about EVD.

To assess behavioral implications, participants were asked if they had changed their behavior as a result of the EVD outbreak, how they would change behavior if an EVD patient were flown from Africa to Germany for treatment in a nearby hospital, and whether they would cancel an already booked flight to Africa. Participants were also asked if they thought that specific prevention measures should be introduced to prevent the spread of EVD to Europe (Figure). The survey also included questions about aid missions in affected countries and about the potential vaccine against EVD.

**Figure:**
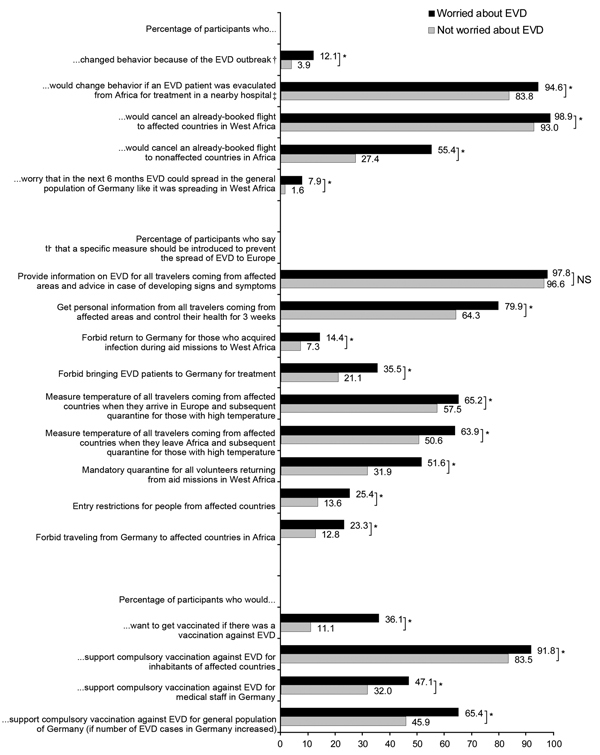
Personal behavior and attitudes toward measures against the spread of Ebola virus disease (EVD) and toward vaccination against EVD. Black, worried about EVD; gray, not worried about EVD; NS, not significant. *χ^2^ test p<0.05. †“Yes” to at least 1 of 5 items (avoid contact with African acquaintances; avoid contact with African persons in public places; avoid going to public events; avoid using public transportation; engage in precautionary purchases). ‡“Yes” to at least 1 of 7 items (avoid public events/crowded places; avoid using public transportation; avoid physical contact with other persons; increase hygiene behavior; wear face mask outside of the home; avoid admission to the same hospital; avoid visiting friends admitted to the same hospital).

This study was approved by the Ethics Committee of the Hannover Medical School and the Federal Commissioner for Data Protection and Freedom of Information. All participants gave written informed consent before entering the study.

### Statistical Analyses

To test differences among groups, we used the χ^2^ test for categorical variables and the Wilcoxon test for continuous variables. To assess associations of sociodemographic factors with worrying about EVD, we used the Spearman correlation coefficient. In addition, we performed explorative multivariable logistic regression analyses to assess the effect of knowledge about transmission routes and sociodemographic factors on worries about EVD and on willingness to volunteer for aid missions. Analyses were performed by using Stata 12 (StataCorp LP, College Station, TX, USA).

## Results

### Risk Perceptions

A total of 974 participants, 15–69 years of age, completed the questionnaire. Sociodemographic characteristics of participants who completed the questionnaire (Table 1) did not differ from those of other panel members who did not (data not shown). In response to the question about whether they worried about EVD, 29% of participants answered in the affirmative; of those, 79.0% rated the strength of their worries as average (score <3 on a scale of 1 [a little] to 5 [very strong]) (data not shown). In response to another question, 68% of the participants reported that they perceived acquiring EVD as possible in at least 1 of the 9 scenarios specified (data not shown). In response to a question asking whether in the next 6 months EVD could spread to the general population of Germany in a similar way as occurred in some West African countries, 8% of participants worried about EVD and 1.6% of those not worried about EVD answered in the affirmative (Figure).

**Table 1:** Characteristics of 974 participants in survey about risk for EVD, Lower Saxony, Germany, 2014*

Characteristic	Total, no. (%)	Not worried about EVD, no. (%)†	Worried about EVD, no. (%)‡	p value§
Sex				0.06
F	534 (54.8)	365 (52.7)	169 (60.1)	
M	414 (42.5)	311 (44.9)	103 (36.7)	
Missing information	26 (2.7)	17 (2.4)	9 (3.2)	
Median age, y (IQR)	46 (34–56)	47 (34–58)	46 (35–54)	0.10¶
Education#				0.02
Low	410 (42.1)	275 (39.7)	135 (48.0)	
Intermediate	117 (12.0)	80 (11.5)	37 (13.2)	
High	411 (42.2)	314 (45.3)	97 (34.5)	
Missing information	36 (3.7)	24 (3.5)	12 (4.3)	
Country of birth				0.59
Germany	891 (91.5)	638 (92.1)	253 (90.1)	
Other	45 (4.6)	30 (4.3)	15 (5.3)	
Missing information	38 (3.9)	25 (3.6)	13 (4.6)	
Median knowledge score (IQR)	7 (6–9)	7 (6–9)	7 (5–8)	<0.001¶

*EVD, Ebola virus disease; IQR, interquartile range. †n = 693 (71.1%). ‡n = 281 (28.9%). §χ^2^ test comparing those worried about EVD with those not worried (missing values were not considered). ¶Wilcoxon rank-sum test comparing those worried about EVD with those not worried.  #Low, <12 y of vocational or secondary education and/or completed apprenticeship; intermediate, at least 12 y of vocational or secondary education and/or degree of a specialized vocational school; high, university training (bachelor degree and higher academic level).

### Knowledge

Although 25% of participants rated their personal knowledge about EVD as good or very good, only 3.9% correctly answered all questions about transmission routes. The most common misperception (by 73.7% of participants) was that airborne transmission of Ebola virus is possible; moreover, 74.0% believed that human-to-human transmission by infected but asymptomatic persons is possible. Among those who specified airborne transmission as being possible, 18.5% reported that they perceived that acquiring EVD while using public transportation was possible compared with 9.4% of those who did not consider airborne transmission as being possible (p = 0.001). Education was positively associated with knowledge scores about Ebola virus transmission routes (Spearman correlation coefficient 0.18, p<0.001) and rating of personal knowledge about EVD (Spearman correlation coefficient 0.39, p<0.001). After controlling for the rating of personal knowledge about EVD, education was no longer associated with the score for knowledge about Ebola virus transmission routes (partial correlation coefficient −0.003, p = 0.91).

### Media Use

Increased use of media to learn about EVD was reported by 43% of participants. These participants most commonly used the Internet (45.5%), television (53.1%), and print media (45.7%). Increased use of television was more common among participants with a low level of vocational or secondary education than among participants with a higher level of education (data not shown). Increased media use was not associated with a higher knowledge score (median score for both groups = 7, p = 0.37). Personal knowledge about EVD was self-rated as good or very good by 28.7% of those who increased their media use and by 21.8% who did not (p = 0.01).

Multivariable logistic regression analyses that included age, sex, education, increased media use, and knowledge score showed that those who increased their media use were more likely to be worried about EVD than were those who did not increase their media use and that knowledge about Ebola virus transmission routes was negatively associated with being worried about EVD (Table 2). Worrying about EVD was not affected by age, sex, or education (Table 2).

**Table 2:** Association between knowledge, media use, sociodemographic factors, and worries about EVD, Lower Saxony, Germany, 2014*

Characteristic	Odds ratio (95% CI)	p value†
Age, per 10-y increase	0.97 (0.87–1.08)	0.52
Sex		0.11
F	1.28 (0.94–1.74)	
M	Reference	
Education‡		0.39
Low	1.28 (0.92–1.78)	
Intermediate	1.33 (0.83–2.14)	
High	Reference	
Increased media use		<0.001
Yes	2.14 (1.59–2.88)	
No	Reference	
Knowledge score (per 1-point increase)	0.87 (0.81–0.93)	<0.001

*Multivariable logistic regression. EVD, Ebola virus disease. †Wald test. ‡Low, <12 y of vocational or secondary education and/or completed apprenticeship; intermediate, at least 12 y of vocational or secondary education and/or degree of a specialized vocational school; high, university training (bachelor degree and higher academic level).

### Personal Reactions

Among all participants, 7% changed behavior in response to the EVD outbreak (Figure). Among those, 68.8% avoided contact with African persons in public places and 26.6% avoided using public transportation.

If an EVD patient were to be flown from Africa to Germany and treated in a nearby hospital, 86.9% of all participants stated that they would change their behavior. Of these, 16.4% would avoid using public transportation, 74.9% would increase their hygiene behavior (e.g., washing hands more often), and 30.2% would not visit friends admitted to the same hospital.

Participants were also asked about travel to Africa. As many as 95% of all participants would cancel an already booked flight to affected countries in West Africa, and 35.6% would cancel a flight to nonaffected countries in Africa.

### Attitudes toward Specific Measures to Prevent the Spread of EVD to Europe

Asked about specific measures to prevent the spread of EVD to Europe, 97.0% of participants replied that all travelers from affected areas should receive information about EVD and advice on what to do if signs and symptoms of EVD developed (Figure). Entry restrictions for persons from affected countries were supported by 17.0% of participants. Mandatory quarantine for volunteers returning from aid missions in West Africa was supported by 37.6% of participants; the difference between those worried about EVD (51.6%) and those not worried (31.9%) was significant (p<0.001). Prohibiting return to Germany of persons who acquired Ebola infection during aid missions was supported by 10%.

### Willingness to Volunteer to Fight EVD in West Africa

Of all participants, 38.7% would volunteer to fight EVD in West Africa if their experience and their knowledge were needed and if their personal situation and their health allowed them to do so. Multivariable logistic regression analyses including age, sex, education, increased media use, and knowledge score showed that older persons were less likely than younger persons to volunteer for aid missions and that women were less likely than men to volunteer (Table 3). Willingness to volunteer was not associated with education level.

**Table 3:** Association between knowledge, media use, and sociodemographic factors and willingness to volunteer in aid missions in Africa, Lower Saxony, Germany, 2014*

Characteristic	Odds ratio (95% CI)	p value†
Age (per 10-y increase)	0.86 (0.77–0.96)	0.07
Sex		0.03
F	0.72 (0.53–0.98)	
M	Reference	
Education‡		0.94
Low	1.09 (0.79–1.52)	
Intermediate	1.05 (0.64–1.70)	
High	Reference	
Increased media use		0.90
Yes	0.98 (0.73–1.32)	
No	Reference	
Knowledge score (per 1-point increase)	1.06 (0.99–1.14)	0.09

*Multivariable logistic regression. EVD, Ebola virus disease. †Wald test. ‡Low, <12 y of vocational or secondary education and/or completed apprenticeship; intermediate, at least 12 y of vocational or secondary education and/or degree of a specialized vocational school; high, university training (bachelor degree and higher academic level).

### Vaccination against EVD

If a vaccine against EVD existed, 18.3% would opt for vaccination even if they did not plan to visit affected countries in West Africa and did not have contact with EVD patients. Of those who wanted to get vaccinated, 41.1% would still do so if the vaccine were associated with occasional mild side effects and 15.2% if it were associated with rare but severe side effects.

Of all participants, 85.9% stated that compulsory vaccination against EVD should be implemented in affected countries. A total of 36.4% would support compulsory vaccination against EVD for medical staff in Germany, and 51.5% would support compulsory vaccination against EVD for the general population of Germany if the number of EVD cases in Germany increased.

## Discussion

We report public perceptions of EVD in Germany, a country not directly affected by the current epidemic. Among the participants of our study, a substantial proportion were worried about EVD; however, among those worried, most did not report strong worries. Only one quarter of participants rated their knowledge of Ebola as good or very good. In addition, a large majority had poor knowledge about the transmission routes of the virus. A particularly common misperception was that Ebola virus can be transmitted by the airborne route or that it can be transmitted from human to human by infected but asymptomatic persons. These misperceptions were strongly associated with perceived personal probability of becoming infected while using public transportation. At the peak of the epidemic (November 2014), we identified inappropriate, unjustified, and stigmatizing attitudes in only a small proportion of participants. In contrast, treatment of a patient flown from Africa to a nearby hospital would induce worrying and inappropriate behavior in most participants. This response might be attributable to the fact that persons intuitively overestimate the risk for rare events (*18*). Our findings indicate a potential for inappropriate reactions to the epidemic should cases of EVD occur in Germany or should evacuations of EVD patients to Germany increase (*19*). For either of these 2 scenarios, trusted institutions (e.g., government) should spread information on the cause and the risk for infection (*20*).

As expected, participants who were worried about EVD were more likely to support measures preventing its spread to Europe. The difference between those worried and those not worried was particularly large for measures that can be considered inappropriate or even counterproductive to fighting the epidemic. For example, the stigmatization of returning health care workers and other volunteers can lead to fewer persons being willing to volunteer for aid missions (*21*). It is crucial that those worried about EVD remain a minority so that society will not be paralyzed by worries. Thus, misperceptions regarding transmission routes of Ebola virus should be resolved, and the media should contribute to a balanced, rational response rather than fuel worries. The observation that increased media use was not associated with better knowledge of transmission routes indicates the need for qualitative improvement of media reporting of such situations. However, the direction of the association between increased media use and worries cannot be determined from our data, so conclusions on worries and increased media use should be made cautiously. Not only the media but also public health experts might have contributed to mixed messages regarding airborne transmission of Ebola virus (*22*).

Almost 39% of participants indicated that they would volunteer to fight EVD in West Africa, but some of those participants would at the same time support prevention measures that are likely to negatively affect willingness to participate in aid missions. The high percentage of volunteers might result from the specific question that the participants were asked. The question included 2 preconditions that would qualify persons to volunteer: having the required experience and having a personal situation that would enable going to Africa. Most participants probably did not fulfill these preconditions, so their willingness to volunteer was only hypothetical. Therefore, they might not have realized that the restriction regarding return of volunteers would hamper their own return.

The changes in personal daily behavior reported or forecasted by the study participants (change of contact structure and mode of transportation, support of rapidly introduced vaccines) have consequences for understanding future emerging epidemics. Mathematical models constructed on the basis of contact structures and health perceptions obtained outside an epidemic setting will not be able to provide helpful insights if they do not take these factors into account. Problems in modeling the further course of the influenza A(H1N1)pdm09 outbreak might be attributable to these factors (*1*,*2*), and the experience with models made for the current EVD epidemic might be similar.

A large majority of participants supported compulsory vaccination against EVD for persons in affected countries. About half also stated that EVD vaccination should be compulsory for the general population should the number of cases in Germany increase. This finding is astonishing because no compulsory vaccination exists in Germany, and during the 2009 influenza A(H1N1)pdm09 pandemic, it was regarded as completely unacceptable (*23*). It is possible that the acceptability of drastic and compulsory measures is high only if the likelihood that such measures will be implemented is low, as is now the situation for EVD. On the contrary, the perception of associated risks might be scored much higher for EVD than for influenza, thereby increasing the acceptance of compulsory vaccination.

This study has some limitations. Regional data collected in an online survey might not represent perceptions of the general population in Germany. Furthermore, because the respondents in our survey were participating in a study on hygiene and behavior regarding infectious diseases, their level of motivation and knowledge about health-related topics might be higher than that of the general population. The education level of participants was also higher than that of the general population (42.2% of the study participants had university training compared with only 17.2% of the general population of Germany; *24*). The panel members were also older, and the percentage of female panel members was higher than that of the general population.

For some characteristics in our analyses we did not have baseline data. For example, we did not have baseline information about which types of media are generally used by participants, so we cannot tell whether participants increased their media use or whether they used additional media sources that they did not use before. Because we do not have information about participants’ professions, we cannot assess whether risk groups for exposure to EVD (e.g., medical staff) are overrepresented in the study sample.

The reported risk perceptions and attitudes are conditional for the situation in Germany as of November 2014 and assume no transmission of Ebola virus in Germany. In the case of real exposure, persons might not act as they predicted they would. We also cannot assess how much the responses are influenced by the current status and how persons would react when media attention is less. However, having access to the study population of the larger infectious diseases study will enable us to ask the same persons again several months later and to examine temporal changes of risk perceptions.

In conclusion, a substantial proportion of the study population demonstrated poor knowledge about the transmission modes of Ebola virus and about the actual risks in a European country during the 2014 EVD epidemic in West Africa. Increased media use was not associated with better knowledge, underscoring the need to improve quality of content reported by the media. Although inappropriate or unjustified attitudes in the current situation were not demonstrated by most participants, the treatment of flown-in EVD patients in a nearby hospital would trigger inappropriate behavioral changes.

**Technical Appendix.** Questionnaire used in online survey of risk perceptions regarding Ebola virus disease, Lower Saxony, Germany, 2014. (German version translated into English, not validated.)
