# Supplementary material for: Ebola Risk Perception in Germany, 2014
Source: Emerg Infect Dis. 2015 Jun;21(6):1012–8. doi: 10.3201/eid2106.150013 (PMC4451905; doi:10.3201/eid2106.150013)
Supplement: Supplementary file 1 — Technical Appendix. Questionnaire used in online survey of risk perceptions regarding Ebola virus disease, Lower Saxony, Germany, 2014. (German version translated into English, not validated.) [file 15-0013-Techapp-s1.pdf]

Article DOI: http://dx.doi.org/10.3201/eid2106.150013

Ebola Risk Perception in Germany, 2014

Technical Appendix

Questionnaire on Ebola

In March 2014, the first cases of Ebola virus disease were notified in Guinea (West Africa). In the following months, the disease spread to Sierra Leone, Liberia, Nigeria, and Senegal. The WHO declared this outbreak as a Public Health Emergency of International Concern.

We are interested in your thoughts about this outbreak and if/how Ebola affects your daily life in Germany.

Ebola virus disease

1. Are you worried about Ebola?

☐ Yes ☐ No

2. *[if yes]* **How much are you worried about Ebola?**

Please mark a number from 1=“not much “ to 5=“very much“

	1	2	3	4	5	
not much	☐	☐	☐	☐	☐	very much

	Very poor	Poor	Moderate	Good	Very good
3. How do you rate your personal knowledge about Ebola virus disease?	☐	☐	☐	☐	☐
4. How do you rate the information that you get from the media about the situation in African countries affected by Ebola?	☐	☐	☐	☐	☐

Probability of acquiring Ebola

5. How can Ebola be transmitted?

	Yes	No	Don't know
By direct contact with bodily fluids of infected persons, either dead or living	☐	☐	☐
By direct contact with infected, but asymptomatic persons	☐	☐	☐
Through air, if infected people cough or sneeze	☐	☐	☐

	Yes	No	Don't know
Through material which has been heavily contaminated with bodily fluids of dead or living infected persons	☐	☐	☐
Through drinking water	☐	☐	☐
Through food produced in Germany	☐	☐	☐
By casual contact with someone already sick, such as sitting next to the person (without any direct contact of bodily fluids)	☐	☐	☐
By wild animals in Africa (monkeys, bats)	☐	☐	☐
By wild animals in Germany (rats, foxes)	☐	☐	☐
By insects in Africa (mosquitoes, tsetse flies)	☐	☐	☐
By insects in Germany (midges)	☐	☐	☐

6. If you think of the recent worldwide situation about Ebola: Do you think that you have a personal risk of acquiring Ebola…

	Highly unlikely	Quite unlikely	Quite likely	Highly likely	Does not apply
… at work?	☐	☐	☐	☐	☐
... in public transport?	☐	☐	☐	☐	☐
... in public places (school, childcare …) or public events?	☐	☐	☐	☐	☐
… at an airport in Germany?	☐	☐	☐	☐	☐
… as a patient in a German hospital?	☐	☐	☐	☐	☐
… at a doctor's office in Germany?	☐	☐	☐	☐	☐
… during a travel to affected countries?	☐	☐	☐	☐	☐
… by food imported from Western African countries?	☐	☐	☐	☐	☐
... by other products originating in West Africa?	☐	☐	☐	☐	☐

7. **Are you worried that…**

	☐Highly unlikely	☐Quite unlikely	☐Quite likely	☐Highly likely
… <u>in the next three months</u> people might arrive in Germany who are identified as infected persons after their entry?	☐	☐	☐	☐
… individual persons might be infected with the Ebola virus in Germany <u>during the next six months</u>?	☐	☐	☐	☐
… <u>in the next six months</u> Ebola could spread in the general population of Germany similar to how it is spreading currently in West Africa?	☐	☐	☐	☐

Personal behavior and prevention measures

8. **Imagine that you have booked a flight for the coming week <u>to travel to any of the affected countries in West Africa</u>. What would you do?**

☐ Take the flight.

☐ Cancel the flight, but only if I am paid back my money.

☐ Cancel the flight even if 100% of the travel costs would be lost.

☐ Cancel the flight, but only to a maximum loss of ☐☐ % of the travel costs.

9. Imagine that you have booked a flight for the coming week <u>to a non-affected country in Africa</u>. What would you do?

☐ Take the flight.

☐ Cancel the flight, but only if I am paid back my money.

☐ Cancel the flight even if 100% of the travel costs would be lost.

☐ Cancel the flight, but only to a maximum loss of ☐☐ % of the travel costs.

10. Did you change your behavior because of the Ebola outbreak in West Africa?

	Yes	No
I engaged in precautionary purchases.	☐	☐
I avoid contact to African people <u>from acquaintanceship</u>.	☐	☐
I avoid contact to African people <u>in public places</u>.	☐	☐
I avoid going to public events (football, theater …).	☐	☐

11. Would you change your behavior if an Ebola patient was evacuated from Africa and brought to Germany for treatment in a near-by hospital?

	No	Rather no	Rather yes	Yes
I would avoid public events and crowded places.	☐	☐	☐	☐
I would avoid using public transport.	☐	☐	☐	☐
I would avoid physical contact with other people.	☐	☐	☐	☐
I would increase my hygiene behavior (e.g. wash my hands more often)	☐	☐	☐	☐
I would wear a face mask outside of my home.	☐	☐	☐	☐
I would not want to be admitted to the same hospital.	☐	☐	☐	☐
I would not visit friends admitted to the same hospital.	☐	☐	☐	☐

12. Should the following measures be introduced to prevent the spread of Ebola to Europe?

	Not on any account	Not encouraged	Encouraged	Yes, absolutely
Provide information on Ebola to all travelers coming from affected areas and provide advice in case one develops signs and symptoms	☐	☐	☐	☐
Get personal information of all travelers coming from affected areas and control their health for three weeks long upon arrival	☐	☐	☐	☐
Forbid return transport for Germans who get infected during aid missions in West Africa	☐	☐	☐	☐
Forbid bringing Ebola patients for treatment to Germany	☐	☐	☐	☐

13. Should the following measures be introduced to prevent the spread of Ebola to Europe?

	Not on any account	Rather no	Rather yes	Yes, absolutely
Measure temperature for all travelers coming from affected countries upon arrival at Europe with subsequent quarantine for those with high temperature	☐	☐	☐	☐
Measure temperature for all travelers coming from affected countries when they are about to leave Africa with subsequent quarantine for those with high temperature	☐	☐	☐	☐
Three weeks of mandatory quarantine for all volunteers returning from aid missions in West Africa	☐	☐	☐	☐

14. Should the following measures be introduced to prevent the spread of Ebola to Europe?

	Not on any account	Rather no	Rather yes	Yes, absolutely
Entry restrictions for people from affected countries	☐	☐	☐	☐
Forbid travelling from Germany to affected countries in Africa	☐	☐	☐	☐
Compulsory vaccination against Ebola for all inhabitants of affected countries as soon as a vaccine is available	☐	☐	☐	☐

Personal commitment

15. Would you volunteer to fight EVD in West Africa if your experience and knowledge were needed and if your personal situation and your health allowed so?

☐ Yes ☐ Likely ☐ Don’t know

☐ Unlikely ☐ No

16. What would be the <u>main</u> reason not to volunteer?

☐ I would be worried about getting infected.

☐ I think that the help would not be useful.

☐ I would be worried that I might not return to Germany if I get infected.

☐ I would be worried about not being able to get back to Germany because of an entry restriction.

☐ I think every country should solve its problems by itself without depending on help from other countries.

17. Would you support a nonrecurring, compulsory, and income-related payment for the fight against Ebola in Africa?

☐ Yes ☐ Likely ☐ Don’t know

☐ Unlikely ☐ No

18. *[if not no]* **Which sum would you pay for the cause?**

☐ Up to 10€

☐ 11 to 20€

☐ 21 to 50€

☐ 51 to 100€

☐ 101 to 200€

☐ More than 200€

Vaccination

19. If a vaccine against Ebola existed, would you opt for the vaccination even if you do not plan to visit affected countries in West Africa and do not have contact with Ebola patients ever?

☐ Yes ☐ No ☐ Don't know

20. *[if not no]* **Would you still do so if the vaccine was associated with occasional mild side effects?**

☐ Yes ☐ No ☐ Don't know

21. *[if not no]* **Would you still do so if the vaccine was associated with rare and severe side effects?**

☐ Yes ☐ No ☐ Don't know

22. Should there be a compulsory vaccination against Ebola for the medical staff in Germany?

☐ Yes ☐ No ☐ Don’t know

23. Should there be a compulsory vaccination against Ebola for the general population in Germany if the number of EVD cases in Germany increased?

☐ Yes ☐ No ☐ Don’t know

24. A vaccine must be tested for safety and efficacy. In which country should this be done?

	Yes	No	Don’t know
In the country that develops the vaccine	☐	☐	☐
In the affected countries of West Africa	☐	☐	☐

Activities about Ebola

25. Have you changed your media use since the Ebola outbreak has become public?

	Yes	No
I use the Internet more often/in addition to inform myself about Ebola.	☐	☐
I use television more often/in addition to inform myself about Ebola.	☐	☐
I use the radio more often/in addition to inform myself about Ebola.	☐	☐
I use print media more often/in addition to inform myself about Ebola.	☐	☐
I use information from public institutions (e.g. Robert Koch-Institute, World Health Organization) more often/in addition to inform myself about Ebola.	☐	☐

26. Did you do something among the following list of actions in connection to Ebola?

Multiple choice

☐ Write a letter to the editor or a blog entry about Ebola in a paper or on the Internet

☐ Write a comment to an article in the Internet (e.g. Tagesschau.de, Spiegel online)

☐ Discussion in the circle of acquaintances

☐ Adhered to preventive measures at work

☐ Donations

☐ Participation in information events

☐ Organizing an information event

☐ Offer to participate in aid missions in Africa

☐ Offer to help in Germany

27. At your workplace, do you have contact to…?

	Yes	No
… patients or persons in need of care?	☐	☐
… more than 10 persons a day (e.g. students, customers, colleagues)?	☐	☐

Submit

Thank you for your answers!

Get information about Ebola:

- http://www.bzga.de/?sid=1295
- www.rki.de/ebola
- http://www.auswaertiges-amt.de/cae/servlet/contentblob/675054/publicationFile/196334/Ebola_Westafrika.pdf

Umfrage zu Ebola

Im März 2014 wurde aus Guinea (West-Afrika) ein gehäuftes Auftreten der Ebola-Virus-Erkrankung berichtet. Die Krankheit breitete sich in den folgenden Monaten nach Sierra Leone, Liberia, Nigeria und in den Senegal aus und wird von der Weltgesund-heitsorganisation als Bedrohung der internationalen Gesundheit eingestuft.

Wir interessieren uns dafür, was Sie über diesen Ausbruch denken und wie/ob Ebola Ihren Alltag in Deutschland beeinflusst.

Ebola-Virus-Erkrankung

7. Haben Sie persönlich Angst vor Ebola?

☐ Ja ☐ Nein

8. *[falls ja]* **Wie stark ist Ihre Angst vor Ebola?**

Bitte kreuzen Sie eine Zahl von 1 = „gering" bis 5 = „sehr stark" an

	1	2	3	4	5	
gering	☐	☐	☐	☐	☐	sehr stark

	Nicht gut	Weniger gut	Mittelmäßig	Gut	Sehr gut
9. Wie schätzen Sie Ihr Wissen über die Ebola-Virus-Erkrankung ein?	☐	☐	☐	☐	☐
10. Wie gut fühlen Sie sich von Medien informiert über die Situation in den von Ebola betroffenen afrikanischen Ländern?	☐	☐	☐	☐	☐

Ansteckungsgefahr

11. Wie kann das Ebola-Virus übertragen werden?

	Ja	Nein	Weiß nicht
Über direkten körperlichen Kontakt zu Ebola-Erkrankten oder -Verstorbenen	☐	☐	☐
Über direkten körperlichen Kontakt zu Personen, die sich mit Ebola angesteckt haben, aber noch keine Symptome zeigen	☐	☐	☐
Durch die Luft, wenn Ebola-Erkrankte niesen oder husten	☐	☐	☐

	Ja	Nein	Weiß nicht
Durch Gegenstände, die mit Körperflüssigkeiten von Ebola-Erkrankten in Kontakt gekommen sind, z. B. Kleidung, Bettwäsche, Besteck	☐	☐	☐
Durch Trinkwasser	☐	☐	☐
Durch in Deutschland produzierte Lebensmittel	☐	☐	☐
Bei gemeinsamem Aufenthalt mit Ebola-Erkrankten in einem Raum, z.B. Busfahrt, ohne direkten körperlichen Kontakt	☐	☐	☐
Durch wildlebende Säugetiere in Afrika (Affen, Fledermäuse)	☐	☐	☐
Durch wildlebende Säugetiere in Deutschland (Ratten, Füchse)	☐	☐	☐
Durch Insekten in Afrika (Moskitos, Tsetsefliegen)	☐	☐	☐
Durch Insekten in Deutschland (Mücken)	☐	☐	☐

12. Wenn Sie an die aktuelle weltweite Situation bezüglich Ebola denken: Befürchten Sie, dass Sie sich mit Ebola infizieren könnten…

	Nein	Eher nein	Eher ja	Ja
… an Ihrem Arbeitsplatz?	☐	☐	☐	☐
… in öffentlichen Verkehrsmitteln (Bus, Bahn etc.)?	☐	☐	☐	☐
… in öffentlichen Einrichtungen (Schule, Kindergarten, …) oder bei öffentlichen Veranstaltungen?	☐	☐	☐	☐
… am Flughafen in Deutschland?	☐	☐	☐	☐
… als Patient in einem deutschen Krankenhaus?	☐	☐	☐	☐
… in einer Arztpraxis in Deutschland?	☐	☐	☐	☐
… bei einer für dieses Jahr geplanten Urlaubsreise oder Dienstreise in Länder mit Erkrankungsfällen?	☐	☐	☐	☐
… durch Lebensmittel aus West-Afrika?	☐	☐	☐	☐
… durch andere Produkte aus West-Afrika (Schnitzereien, Schalen, …?	☐	☐	☐	☐

13. Befürchten Sie, dass…

	Nein	Eher nein	Eher ja	Ja
… <u>in den nächsten 3 Monaten</u> Personen nach Deutschland einreisen, bei denen erst nach Einreise auffällt, dass sie angesteckt sind?	☐	☐	☐	☐

	Nein	Eher nein	Eher ja	Ja
… sich in Deutschland <u>in den nächsten 6 Monaten</u> vereinzelt Personen mit dem Ebola-Virus anstecken und erkranken?	☐	☐	☐	☐
… sich das Ebola-Virus in Deutschland <u>in den nächsten 6 Monaten</u> in der allgemeinen Bevölkerung so verbreitet wie jetzt in den betroffenen Ländern in West-Afrika?	☐	☐	☐	☐

Persönliches Verhalten und Schutzmaßnahmen

14. Wenn Sie für die nächste Woche eine Urlaubsreise <u>in die betroffenen Gebiete in West-Afrika</u> gebucht hätten, was würden Sie tun?

☐ Die Reise antreten.

☐ Die Reise stornieren, aber nur, wenn die Kosten erstattet werden würden.

☐ Die Reise stornieren, auch wenn 100% des gezahlten Reisepreises verfallen würden.

☐ Die Reise stornieren, aber nur bis zu einem Verlust von ☐☐ % des Reisepreises.

15. Wenn Sie für die nächste Woche eine Urlaubsreise <u>in nicht betroffene Teile von Afrika</u> gebucht hätten, was würden Sie tun?

☐ Die Reise antreten.

☐ Die Reise stornieren, aber nur, wenn die Kosten erstattet werden würden.

☐ Die Reise stornieren, auch wenn 100% des gezahlten Reisepreises verfallen würden.

☐ Die Reise stornieren, aber nur bis zu einem Verlust von ☐☐ % des Reisepreises.

16. Haben Sie Ihr Verhalten seit dem Bekanntwerden des Ebola-Ausbruchs in West-Afrika verändert?

	Ja	Nein
Ich habe Lebensmittel auf Vorrat eingekauft.	☐	☐
Ich meide <u>in meinem Bekanntenkreis</u> den Kontakt zu Personen, die aus Afrika stammen.	☐	☐
Ich meide <u>an öffentlichen Plätzen</u> den Kontakt zu Personen, die aus Afrika stammen.	☐	☐
Ich gehe nicht mehr zu öffentlichen Veranstaltungen (Fußballspiele, Theater, …).	☐	☐

17. Würden Sie Ihr Verhalten ändern, wenn in einem Krankenhaus in Ihrer Nähe auf der Isolierstation ein aus Afrika eingeflogener Ebola-Patient liegen würde?

	Nein	Eher nein	Eher ja	Ja
Ich würde öffentliche Plätze/Veranstaltungen oder Menschenansammlungen meiden.	☐	☐	☐	☐
Ich würde öffentliche Verkehrsmittel meiden.	☐	☐	☐	☐
Ich würde Körperkontakt meiden (anderen Personen nicht die Hand geben etc.).	☐	☐	☐	☐
Ich würde stärker auf Hygiene achten (häufigeres Händewaschen o.Ä.).	☐	☐	☐	☐
Ich würde außerhalb meiner Wohnung eine Atemschutzmaske tragen.	☐	☐	☐	☐
Ich würde nicht in dasselbe Krankenhaus eingewiesen werden wollen.	☐	☐	☐	☐
Ich würde Freunde/Angehörige, die im selben Krankenhaus liegen, nicht besuchen.	☐	☐	☐	☐

18. Sollten folgende Maßnahmen eingesetzt werden, um eine Ausbreitung der Ebola-Epidemie nach Europa zu vermeiden?

	Nein, auf keinen Fall	Eher nein	Eher ja	Ja, unbedingt
Verteilen von speziellen Informationen am Flughafen an alle Einreisenden aus den betroffenen afrikanischen Ländern, auf welche Krankheitszeichen sie bei sich achten sollen und wohin sie sich bei Auftreten von Symptomen wenden sollen	☐	☐	☐	☐
Namentliche Erfassung aller einreisenden Flugpassagiere aus betroffenen afrikanischen Ländern und 3-wöchige Gesundheitskontrolle durch Gesundheitsämter in Deutschland	☐	☐	☐	☐
Verbot des Rücktransports für in Afrika hilfeleistende Deutsche, die dort an Ebola erkrankt sind	☐	☐	☐	☐
Einreiseverbot für Bürger aus betroffenen afrikanischen Ländern, die zur Behandlung einreisen würden	☐	☐	☐	☐

19. Sollten folgende Maßnahmen eingesetzt werden, um eine Ausbreitung der Ebola-Epidemie nach Europa zu vermeiden?

	Nein, auf keinen Fall	Eher nein	Eher ja	Ja, unbedingt
Messung der Körper-Temperatur bei allen einreisenden Flugpassagieren aus betroffenen afrikanischen Ländern <u>bei Ankunft in Europa</u> mit anschließender 3-wöchiger Isolierung für Personen mit erhöhter Temperatur	☐	☐	☐	☐
Messung der Körper-Temperatur bei allen ausreisenden Flugpassagieren aus betroffenen afrikanischen Ländern <u>beim Abflug in Afrika</u> mit anschließender 3-wöchiger Isolierung im Abflugland und Einreiseverbot für Personen mit erhöhter Temperatur	☐	☐	☐	☐
3-wöchige Isolierung nach Einreise in Deutschland für alle Personen, die in Afrika Hilfe geleistet haben und dann nach Deutschland zurückkehren, auch wenn kein Verdacht auf Ebola besteht				

20. Sollten folgende Maßnahmen eingesetzt werden, um eine Ausbreitung der Ebola-Epidemie nach Europa zu vermeiden?

	Nein, auf keinen Fall	Eher nein	Eher ja	Ja, unbedingt
Verbot der Einreise aus betroffenen afrikanischen Ländern (keine Vergabe von Visa), auch wenn kein Verdacht auf Ebola besteht	☐	☐	☐	☐
Verbot der Ausreise aus Deutschland in betroffene afrikanische Gebiete	☐	☐	☐	☐
Impfplicht für alle Bewohner der betroffenen afrikanischen Gebiete, sobald ein Impfstoff verfügbar ist	☐	☐	☐	☐

Persönlicher Einsatz

21. Angenommen, Ihre Erfahrung und Ihr Wissen würden gebraucht, um in Afrika vor Ort Hilfeleistungen zu erbringen: Würden Sie sich zur Verfügung stellen, wenn Ihre persönliche Situation es zuließe?

☐ Ja ☐ Eher ja ☐ Weiß nicht
☐ Eher nein ☐ Nein

22. Was wäre für Sie der wichtigste Grund, sich nicht zur Verfügung zu stellen?

☐ Ich hätte Angst vor Ansteckung.
☐ Ich glaube, dass die Hilfe nicht sinnvoll ist.
☐ Ich hätte Angst, im Falle einer Erkrankung nicht nach Deutschland zurückgebracht werden zu können.
☐ Ich hätte Angst, nicht nach Deutschland zurückkehren zu dürfen (in dem Fall, dass die Grenzen vollständig geschlossen werden würden).
☐ Ich glaube, jedes Land sollte seine Probleme selbst lösen ohne Helfer aus dem Ausland.

23. Angenommen, in Deutschland solle eine einmalige, verpflichtende, einkommensabhängige Abgabe zur Bekämpfung von Ebola in Afrika erhoben werden: Würden Sie dies befürworten?

☐ Ja ☐ Eher ja ☐ Weiß nicht

☐ Eher nein ☐ Nein

24. *[falls nicht nein]* **Welchen Betrag wären Sie bereit, in diesem Rahmen zur Verfügung zu stellen?**

☐ Bis zu 10 €

☐ 11 bis 20 €

☐ 21 bis 50 €

☐ 51 bis 100 €

☐ 101 bis 200 €

☐ Mehr als 200 €

Impfung

25. Wenn es eine zugelassene Impfung gegen Ebola gäbe, würden Sie sich vorbeugend impfen lassen (auch wenn Sie nicht vorhaben, in betroffene Gebiete in West-Afrika zu reisen, und keine beruflichen Kontakte mit Ebola-Patienten haben)?

☐ Ja ☐ Nein ☐ Weiß nicht

26. *[falls nicht nein]* **Würden Sie dies auch machen, wenn bekannt wäre, dass die Impfung gelegentlich Komplikationen auslöst?**

☐ Ja ☐ Nein ☐ Weiß nicht

27. *[falls nicht nein]* **Würden Sie dies auch machen, wenn bekannt wäre, dass die Impfung extrem selten schwere Komplikationen auslöst?**

☐ Ja ☐ Nein ☐ Weiß nicht

28. Sollte Ihrer Meinung nach das medizinische Personal in Deutschland verpflichtend gegen Ebola geimpft werden (wenn es einen zugelassenen Impfstoff gäbe)?

☐ Ja ☐ Nein ☐ Weiß nicht

29. Sollte es Ihrer Meinung nach im Falle steigender Erkrankungszahlen in Deutschland eine Impfpflicht für die deutsche Bevölkerung geben (wenn es einen zugelassenen Impfstoff gäbe)?

☐ Ja ☐ Nein ☐ Weiß nicht

30. Damit ein Impfstoff zugelassen werden kann, muss er zuvor auf Verträglichkeit und Wirksamkeit getestet werden. Wo sollte Ihrer Meinung nach ein Ebola-Impfstoff getestet werden?

	Ja	Nein	Weiß nicht
In dem Land, in dem die Impfung entwickelt wird	☐	☐	☐
In den betroffenen Ländern in West-Afrika	☐	☐	☐

Aktivitäten zu Ebola

31. Hat sich Ihr Medien-Verhalten seit dem Bekanntwerden des Ebola-Ausbruchs verändert?

	Ja	Nein
Ich nutze zusätzlich/vermehrt das Internet, um mich über Ebola zu informieren.	☐	☐
Ich nutze zusätzlich/vermehrt das Fernsehen, um mich über Ebola zu informieren.	☐	☐
Ich nutze zusätzlich/vermehrt das Radio, um mich über Ebola zu informieren.	☐	☐
Ich nutze zusätzlich/vermehrt Printmedien (Zeitungen, Magazine o.Ä.), um mich über Ebola zu informieren.	☐	☐
Ich nutze zusätzlich/vermehrt Informationsangebote öffentlicher Einrichtungen (Bundeszentrale für gesundheitliche Aufklärung, Robert Koch-Institut, Weltgesundheitsorganisation etc.), um mich über Ebola zu informieren.	☐	☐

32. Haben Sie im Zusammenhang mit Ebola etwas aus der folgenden Liste gemacht?

Mehrfachantworten möglich

☐ Schreiben von Leserbriefen/Blog zu dem Thema in Zeitungen oder Internet

☐ Online-Kommentare zu Artikeln im Internet (z.B. bei Spiegel online, Tagesschau.de)

☐ Diskussion im persönlichen Umfeld

☐ Krisen/Notfall/Sicherheitsmaßnahmen am Arbeitsplatz

☐ Spenden

☐ Teilnahme an Informationsveranstaltungen (Vorträge o.Ä.)

☐ Organisation von Informationsveranstaltungen

☐ Angebot, in Afrika persönlich zu helfen

☐ Angebot, von Deutschland aus durch persönlichen Einsatz zu helfen

33. Haben Sie in Beruf oder Freizeit regelmäßig Kontakt zu…?

	Ja	Nein
… Patienten oder pflegebedürftigen Menschen?	☐	☐
… mehr als 10 Personen pro Tag, z.B. Schüler, Kunden, Kollegen etc.?	☐	☐

Absenden

Vielen Dank für Ihre Antworten!

Informationen zum Thema Ebola finden Sie hier:

- http://www.bzga.de/?sid=1295
- www.rki.de/ebola
- http://www.auswaertiges-amt.de/cae/servlet/contentblob/675054/publicationFile/196334/Ebola_Westafrika.pdf
